# Differences in social activity increase efficiency of contact tracing

**DOI:** 10.1140/epjb/s10051-021-00222-8

**Published:** 2021-10-19

**Authors:** Bjarke Frost Nielsen, Kim Sneppen, Lone Simonsen, Joachim Mathiesen

**Affiliations:** 1 Niels Bohr Institute, University of Copenhagen, Blegdamsvej 17, 2100 Copenhagen, Denmark; 2 Department of Science and Environment, Roskilde University, 4000 Roskilde, Denmark

## Abstract

**Abstract:**

Digital contact tracing has been suggested as an effective strategy for controlling an epidemic without severely limiting personal mobility. Here, we use smartphone proximity data to explore how social structure affects contact tracing of COVID-19. We model the spread of COVID-19 and find that the effectiveness of contact tracing depends strongly on social network structure and heterogeneous social activity. Contact tracing is shown to be remarkably effective in a workplace environment and the effectiveness depends strongly on the minimum duration of contact required to initiate quarantine. In a realistic social network, we find that forward contact tracing with immediate isolation can reduce an epidemic by more than 70%. In perspective, our findings highlight the necessity of incorporating social heterogeneity into models of mitigation strategies.

**Graphic abstract:**

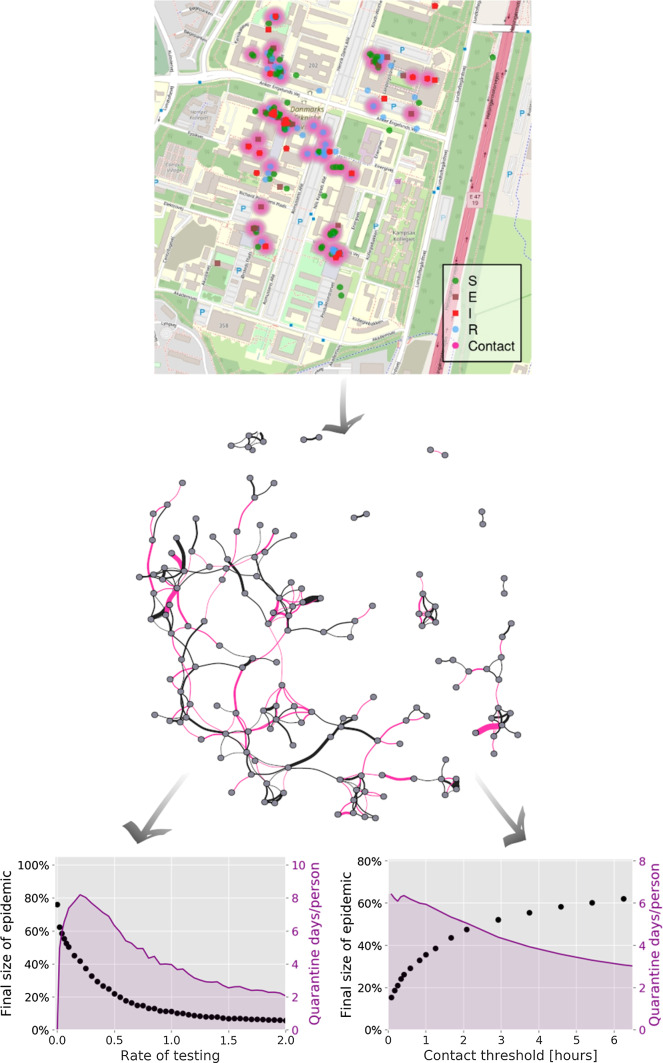

**Supplementary Information:**

The online version supplementary material available at 10.1140/epjb/s10051-021-00222-8.

## Introduction

For diseases which are primarily transmitted in spatial proximity, contact patterns invariably play a central role in the course of an epidemic [1, 2]. For the purposes of modeling infectious diseases, contact patterns can be represented by a network where each individual is a node and spatial proximity between individuals is represented by time-dependent edges. Nonetheless, well-mixed compartmental models remain the typical approach to modeling epidemics [3–6]. Even such models, which do not incorporate a network structure, make assumptions about the underlying social contact patterns. In well-mixed models, the assumption is that mixing patterns are homogeneous *inside* sub-populations [7–13]. Although interaction rates *between* sub-populations can be adjusted, well-mixed models may fail to predict the evolution of an epidemic when social interactions are spatiotemporally restricted [14–16], as in real contact networks.

An oft-taken approach to modelling of contact tracing schemes is branching process simulation [17–19]. In these models, the outbreak is modeled generation by generation and the susceptible population is usually taken to be constant in size, rendering the models most useful for studying early outbreaks. Such models have clear advantages in terms of mathematical tractability, but lack the (disease and social) dynamics which is the main focus of this study. Social interactions tend to follow a characteristic pattern of spatiotemporal correlation, where you meet the same people at specific times during a week, at work or at home. At the same time, social activity varies significantly from person to person. This correlation increases transmission heterogeneity, i.e. the tendency of cases to occur in clusters.

During the COVID-19 pandemic, contact tracing has been the center of much attention due to its promises of epidemic control without severely restricting mobility [20–25]. As a mitigation strategy, contact tracing relies directly on the contact network structure and may benefit from clustering of cases [26]. In order to assess contact tracing strategies, detailed information on contact networks is indispensable, and the usual well-mixed approach is inadequate – more so than when modelling unmitigated spreading [27, 28]. A recent review of models and data of collective dynamics in public safety concluded that central assumptions of classical, homogeneous models of epidemic spread are not even approximately valid in real-world scenarios [29]. Their conclusions further emphasize the importance of utilizing empirical population data in modeling collective dynamics, and of taking existing heterogeneities into account.

In this paper, we utilize Bluetooth proximity data obtained from a cohort of university students at a large European university (see “Methods” for details). In most studies of this nature, mobility data collected from mobile phones rely on spatial locations derived from estimated distances to cell towers, GPS coordinates [30] or the proximity to known Wi-Fi access points. Whereas this kind of data is useful for studies of aggregate mobility [31], the accuracy is typically not sufficient to infer epidemiologically relevant social proximity between individuals. In contrast, the Bluetooth data that we consider here can identify social proximity with a high spatial resolution (< 1 m). In addition, our data has a high temporal resolution (< 5 min), meaning that brief encounters (individuals passing by each other) can be distinguished from longer meetings. A high spatiotemporal resolution is necessary to faithfully simulate disease transmission through a social network, since diseases (such as COVID-19) may be less likely to transmit during short encounters or between individuals separated by more than a few meters [32, 33]. The upper limit for the range of our Bluetooth data is approximately 15 m [34]. We also note that our data are similar in nature to those collected by contact tracing smartphone applications [35].

Like all real-life proximity data, the data set used in this study comprises just a section of the complete contact network of each participant. However, our data still display a well-defined and robust heterogeneity which is the object of our study. We further note that contact heterogeneity is pronounced despite the fact that our participant group is homogeneous in age and occupation, and would be treated as undifferentiated in typical epidemiological models.

To study the effects of contact heterogeneity on an epidemic, we simulate the propagation of COVID-19 on the empirical contact network, and compare with artificially homogenized versions of this network. This allows us to maintain certain features of the network (size, average contact rate) while altering others (network structure and degree distribution). We can then separately study how these features affect the outcomes of the epidemic, with and without mitigation. For that purpose, we introduce three degrees of heterogeneity: (i) the true (observed) network. (ii) an edge swapped version of the network [36], which retains heterogeneity in activity levels but homogenizes the network structure and edge correlations, and (iii) a randomized network, which retains only the overall (mean) contact frequency, but eliminates heterogeneity.

Our main question is if contact tracing of of COVID-19 is affected by the variation in individual social activity levels and by the structure of the social network itself. Our contact tracing algorithm has two key parameters, the probability for a symptomatic individual to undergo testing and the maximum duration of social proximity to an exposed individual allowed, before a self-quarantine is triggered. The latter is especially useful, since it is a directly controllable parameter when e.g. designing contact tracing smartphone applications [35].

**Fig. 1:**
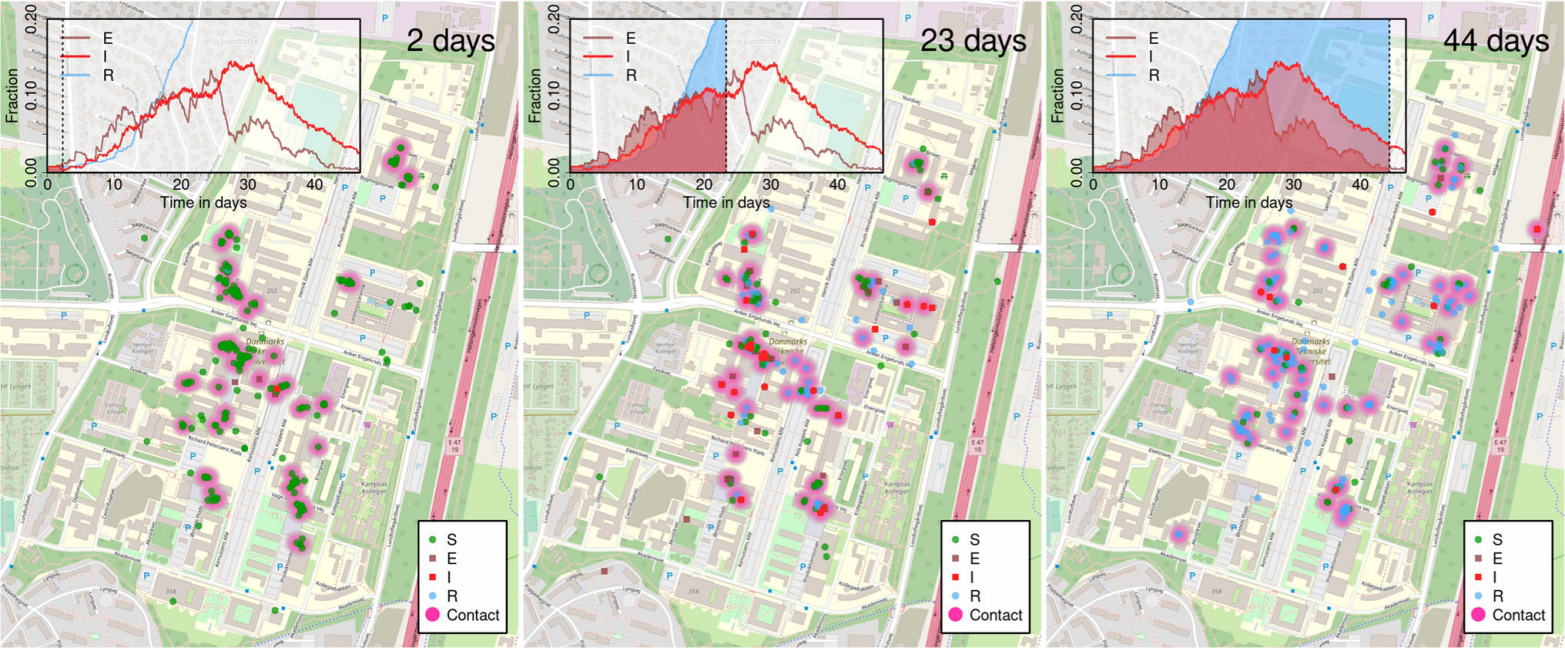
Simulating the spread of COVID-19 on the contact network. Here, a zoom view on the geographical positions of a few individuals (based on GPS coordinates) during a typical work day and for a representative run of the epidemic model. Regions of contact (defined by signal strength exceeding the $-\,85$ dBm cutoff) are shown as diffuse clouds of pink. Snapshots shown are at day 2, 23 and 44 of the outbreak

**Fig. 2:**
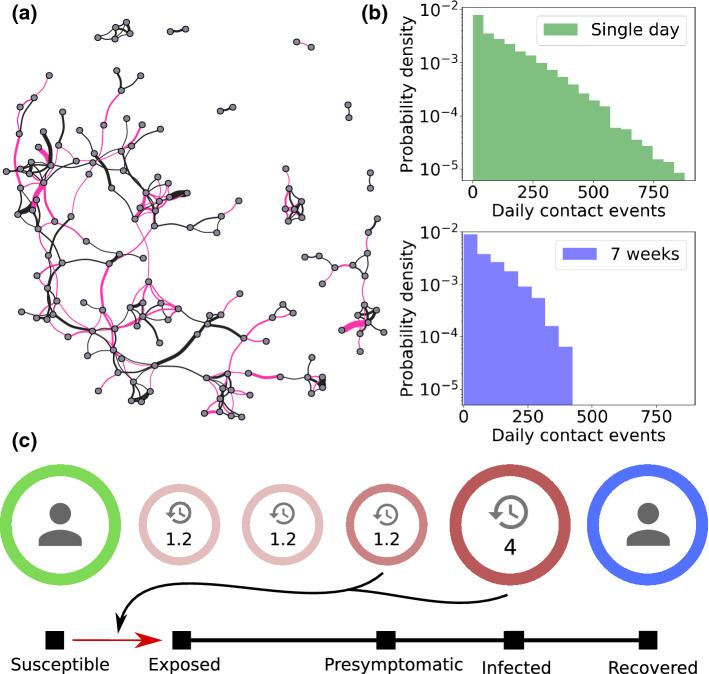
**a** A small subset of a contact network for 1 week. Link thickness indicates the cumulative contact time, with links with less than 2 h cumulative activity being omitted. Black lines represent the links recurring from the previous week, whereas the red lines are new links. **b** Top: histogram of contact events over a single day (semi-logarithmic plot). The coefficient of variation is $c_V = 1.03$ and the mean is $\mu = 131$. Bottom: histogram of contact events over a 7 week period, divided by the number of days to obtain an average daily rate (semi-logarithmic plot). Here, $c_V = 0.95$ and $\mu = 86$. Both plots show a marked heterogeneity, demonstrating that contact heterogeneity is approximately a quenched disorder on the timescale of a few weeks. **c** Our agent-based model of COVID-19 spreading on a contact network. Individuals in the susceptible state may be exposed by those in the presymptomatic as well as infected states. The exposed-presymptomatic triplet of states together comprise the gamma-distributed incubation period

## Methods

We use temporally resolved social proximity data collected using smartphones distributed to 1000 participants (undergraduate students at the Technical University of Denmark [37, 38]). The smartphones were equipped with an application that collected communication in the form of call and text messaging logs, geo-location (GPS coordinates) and social proximity data using the Bluetooth port. Every 5 min, all smartphones in the study scanned for nearby devices included in the study, and recorded Bluetooth signal strength as well as the GPS coordinates of the phone. The data we consider were collected over a period of two years, 2013–2015.

The approximate distance between participants can be inferred from the strength (RSSI) of the Bluetooth signal transmitted between devices. The signal strength can resolve distances in the range of $\le 1$ meter to approximately 10–15 m [34]. To prepare our data for modeling of disease transmission, the collected RSSI values are related to an epidemiologically relevant notion of *contact*. The definition of *a contact* depends on the disease in question and its dominant mode(s) of transmission. If environmental transmission is significant, a simple short-distance cutoff would be incorrect, and simple proximity data would be insufficient. However, for SARS-CoV-2, there is evidence that transmission by fomite is minor [39]. Our transmission model assumes that the transmission risk of COVID-19 increases sharply as interpersonal distance is decreased below 1–2 m [33, 40–43]. Thus, we define two individuals to be in social contact whenever the Bluetooth signal strength between their respective devices exceeds $-\,85$ dBm. This definition of contact captures essentially all $\le 1\text {m}$ interactions while excluding a large portion of the $3\text {m}$ interactions and above [34].

From the social contacts, we can create a well-defined time-dependent contact network where individuals are represented by nodes and social contact by time-dependent links, similar in nature to the network used in [44]. The link activity, i.e. the contact between individuals, is resolved in temporal windows of 5 min. This time-dependent contact network is the basis for our modeling of the transmission of COVID-19.

We model the spread of COVID-19 by an agent-based model (where the study participants serve as the agents) with five states: susceptible to the disease, exposed, pre-symptomatic (but infectious), infected (possibly with symptoms) and recovered/removed. In the absence of contact tracing (described below), the P and I states are identical, in that an individual in one of these states can infect others. Aside from these mutually exclusive states, persons can also be flagged as quarantined. In Fig. 1 an example trajectory is shown, together with a closeup of the university campus. The disease progression model is illustrated in Fig. 2.

The transmission routine works by assuming a constant pairwise infection rate between individuals, when they are in contact. When a susceptible person comes into contact with a person in the **I** or **P** state, there is a probability $p_\text {inf}$ of transmission of the disease in each 5-min window. The basic model (without contact tracing) thus has four parameters: the transmission probability upon contact $p_\text {inf}$, and three time-scales characterizing the exposed, presymptomatic and infected states, $\tau _\text {E}$, $\tau _\text {P}$ and $\tau _\text {I}$.

As shown in Fig. 2, we assume the incubation time to be gamma-distributed with a mean of 3.6 days, of which the last 1.2 days comprise the presymptomatic infectious state. The infectious state, where symptoms may be displayed, is set at 4 days. The last remaining parameter of the disease model, the transmission probability in each window of time, is fitted to reproduce a daily growth rate of 23% in the early epidemic, based on estimates from [45, 46]. This gives a basic reproductive number of $R_0 = 2.8$ when simulated on the empirical social network. Note that this is the pre-mitigation value, which fits well with the reproductive number obtained in a recent review [47].

By employing two different ways of *shuffling* the network connections (edges), we study both the effects of heterogeneity in activity levels (social contact time) and in the network structure. The first method, *edge swapping*, preserves the degree of connectivity of each person (node), while destroying any network structure arising from e.g. group formation and spatial preferences [36]. The second method, *randomization*, preserves only the overall connectivity level in each window of time, but homogenizes the number of contacts for each person.

The edge swapping procedure works as follows. Given a contact network at an instant of time (representing, in our case, a 5 min time window), we iterate the following steps:

- Select two edges at random. Denote the pairs of connected nodes A$\leftrightarrow$B and C$\leftrightarrow$D, respectively.
- Swap the chosen edges such that the connected pairs are now A$\leftrightarrow$C and B$\leftrightarrow$D.

This is repeated until each edge in the system has been swapped several times, on average. Since no node loses or gains an edge by this procedure, the degree distribution is unchanged. Thus, the heterogeneity in social activity levels is preserved as well. However, since edge swapping is performed independently during each time step, the durations of contacts are not preserved. A 10 min contact is thus treated as two 5-min contacts and each undergoes swapping independently. In the supplemental material, we describe a duration-preserving variation on the edge swapping algorithm.

The randomization procedure is simpler, and each iteration proceeds as follows:

- Select an edge at random. Rewire the edge by replacing its endpoints with two nodes, chosen at random from the entire system.

As with the edge swapping procedure, this is repeated until each edge of the network has been swapped several times, on average. Since edges are only rewired, and not created or destroyed, the overall connectivity of the network is preserved.

### Contact tracing

The contact tracing scheme consists of two parts: *regular testing* of symptomatic individuals (with a constant rate of testing $r_\text {test}$) and the contact tracing algorithm itself, which is activated once an individual tests positive. Once a positive individual is found by regular testing, their recent contacts are put in quarantine for a specified time and tested once the quarantine period has elapsed (before potential release). In other words, the contact tracing scheme proceeds as follows:

- For each individual, a list of contact events is kept. When a person (the ‘index case’) is tested positive, all contacts older than 5 days (the *retention time*) are discarded, the index case is quarantined for 5 days.
- If a traced individual has been in contact with the index case for longer than a certain cumulative *contact threshold*, the traced individual is also quarantined for 5 days.
- After the quarantine period has elapsed, the individual is tested. If negative, the individual is released. Otherwise a new 5-day quarantine is issued.

The quarantine is assumed to be instantaneous and a quarantined person is assumed to have no contact with others. We assume that regular testing happens at a constant rate when an individual is in the symptomatic infected state. This rate of testing $r_\text {test}$ is measured in units of $1/\tau _I$, the rate at which an individual leaves the infected state. Thus a rate of testing of e.g. 1 corresponds to a 50% chance of being tested while infected. Note that the simple algorithm used here is non-recursive. This choice was made to simplify the analysis, i.e. to facilitate the comparison of contact tracing in networks with different types of heterogeneity. For an exploration of the impact of recursive vs. standard contact tracing, we refer to Refs. [48, 49].

**Fig. 3:**
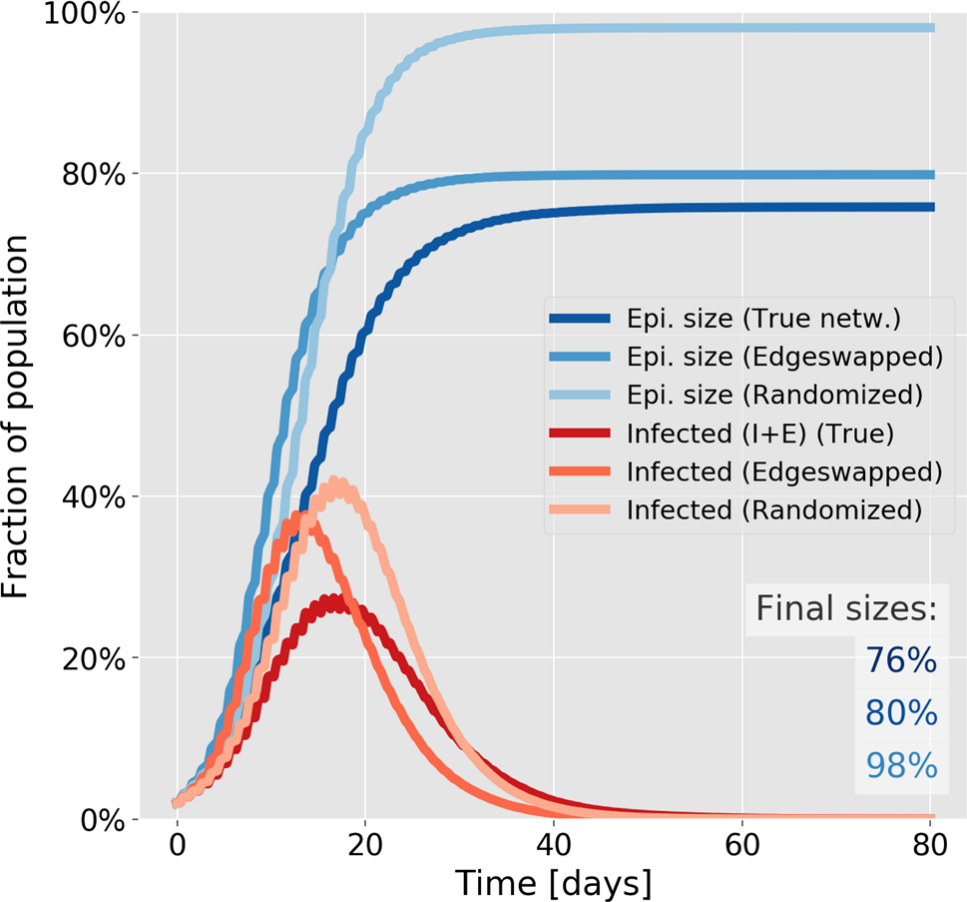
The effects of social heterogeneity on an unmitigated epidemic. The red curves show the incidence, measured as the sum of exposed and infectious individuals (whether symptomatic or not). The blue curves indicate the attack rate, i.e. the cumulative fraction of the population who have been exposed to the disease. In both cases, the curves correspond to the *true*, *edge swapped* and *randomized* networks, in order of increasing brightness. Each trajectory represents an average of 50 simulations

The minimum quarantine time is set at 5 days in our simulations, as suggested by [25], but we have performed a sensitivity analysis (see Supplemental Material) which shows that, while there is still some benefit, the marginal effect of increasing the quarantine time decreases above 5 days. We also performed a sensitivity analysis for the retention time, i.e. the maximum age of contact events deemed relevant when performing contact tracing. It is clear that including contacts which occurred long ago will lead to many unnecessary quarantines, but also that it may increase epidemic control. Our sensitivity analysis shows that the total time spent in quarantine depends only weakly on the retention time, but indicates that 5 days is a reasonable trade-off. See the Supplemental Material for details.

## Results

The distribution of the number of daily contact events for each person in the study is found to closely follow an exponential distribution (Fig. 2b), with a coefficient of variation of 1.03 and a mean of 131. This reflects a marked heterogeneity in activity levels. When we consider the distribution over a 7-week window, a significant degree of contact heterogeneity is retained, albeit with some attenuation. Here the coefficient of variation is 0.95, still close to the value for an exponential distribution, and the mean is 86. It is clear that extreme social behaviour becomes less frequent over the longer time-window, reflecting that individuals do not participate in larger social events every single day. The mean value of 86 corresponds to individuals being socially inactive on 34% of workdays.

**Fig. 4:**
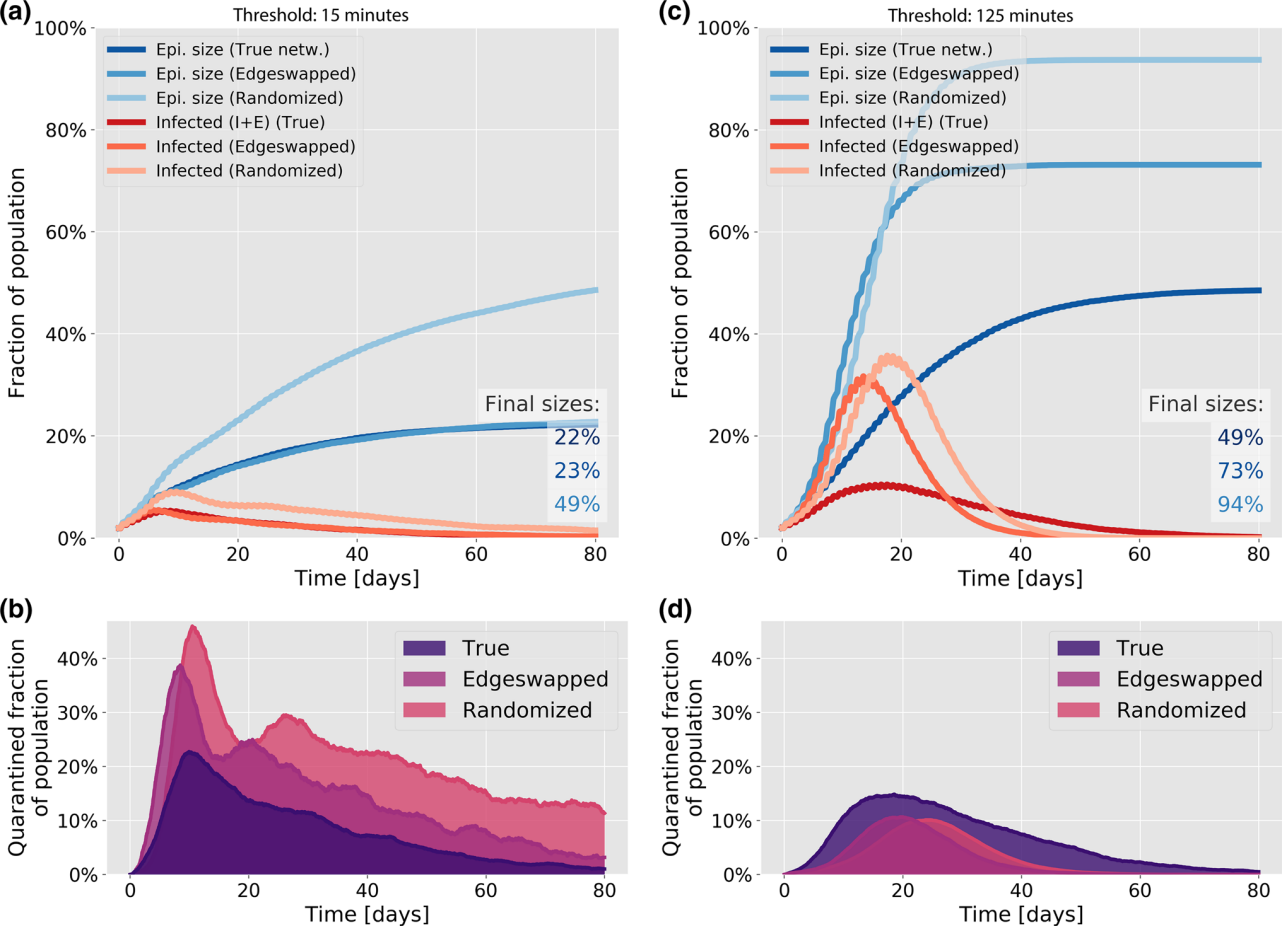
The effects of social heterogeneity on contact tracing at different thresholds. Comparison of exposed + presymptomatic + infected (red) and recovered (blue) individuals in the three networks types. The testing rate is set at 0.5 times the rate for leaving the symptomatic infectious stage, giving a 25% probability of being tested while infected

*Social structure reduces the epidemic severity* To assess the impact of social heterogeneity on an unmitigated epidemic, we compare the simulated evolution of COVID-19 on three different contact networks (Fig. 3): The true (unshuffled) network, the edge swapped and the fully randomized network where each person is assigned an average contact frequency. Each trajectory is averaged over 50 runs, each similar in nature to the one shown in the inserts of Fig. 1.

We find that the final size of the epidemic (the total number of exposed individuals) is very sensitive to heterogeneity in social activity, but not to the network structure. Heterogeneity in social activity prevents the disease from spreading to all parts of the network, with the total fraction exposed reaching 76% in the true network and 98% in the randomized network. The edge swapped network, on the other hand, results in an epidemic size similar to the true network, despite the homogenization of social network structure caused by this procedure.

The epidemic *peak*, on the other hand, is quite sensitive to the social structure. The peak height increases by 10 percentage points when social network structure is destroyed, whereas eliminating the differences in social activity levels as well causes a further increase of just 4 percentage points. Furthermore, the heterogeneous activity leads to a faster initial growth of the epidemic, reaching the peak earlier. The mechanism behind this is that highly socially active individuals are more likely to contract as well as transmit the disease, meaning that they dominate the early epidemic.[50]

*Tracing depends on heterogeneity in a contact threshold-sensitive fashion* Contact tracing is most effective on the true social network, and performs poorly on the randomized network (Fig. 4), regardless of the contact threshold. The relative efficiency on the edge swapped network, however, depends quite strongly on the contact threshold, i.e. on how much cumulative contact time with a known infected person is allowed before triggering a quarantine. With a fairly short contact threshold of 15 min (Fig. 4a), contact tracing on the edge swapped and true network are both highly effective, resulting in a final epidemic sizes of 22–23%. With a higher contact threshold of 125 min (Fig. 4c, d), contact tracing is much less effective in general, but now both of the homogenized networks perform much worse than the true network. This finding owes to the fact that repeated contacts are less frequent in the homogenized networks. It also explains the fact that the average quarantine time is much lower in the homogenized networks at high thresholds (Fig. 4d), since very few infected contacts are traced. A higher contact threshold thus has the advantage of reducing the overall time spent in quarantine (Fig. 4b, d) but results in a reduced epidemic control.

**Fig. 5:**
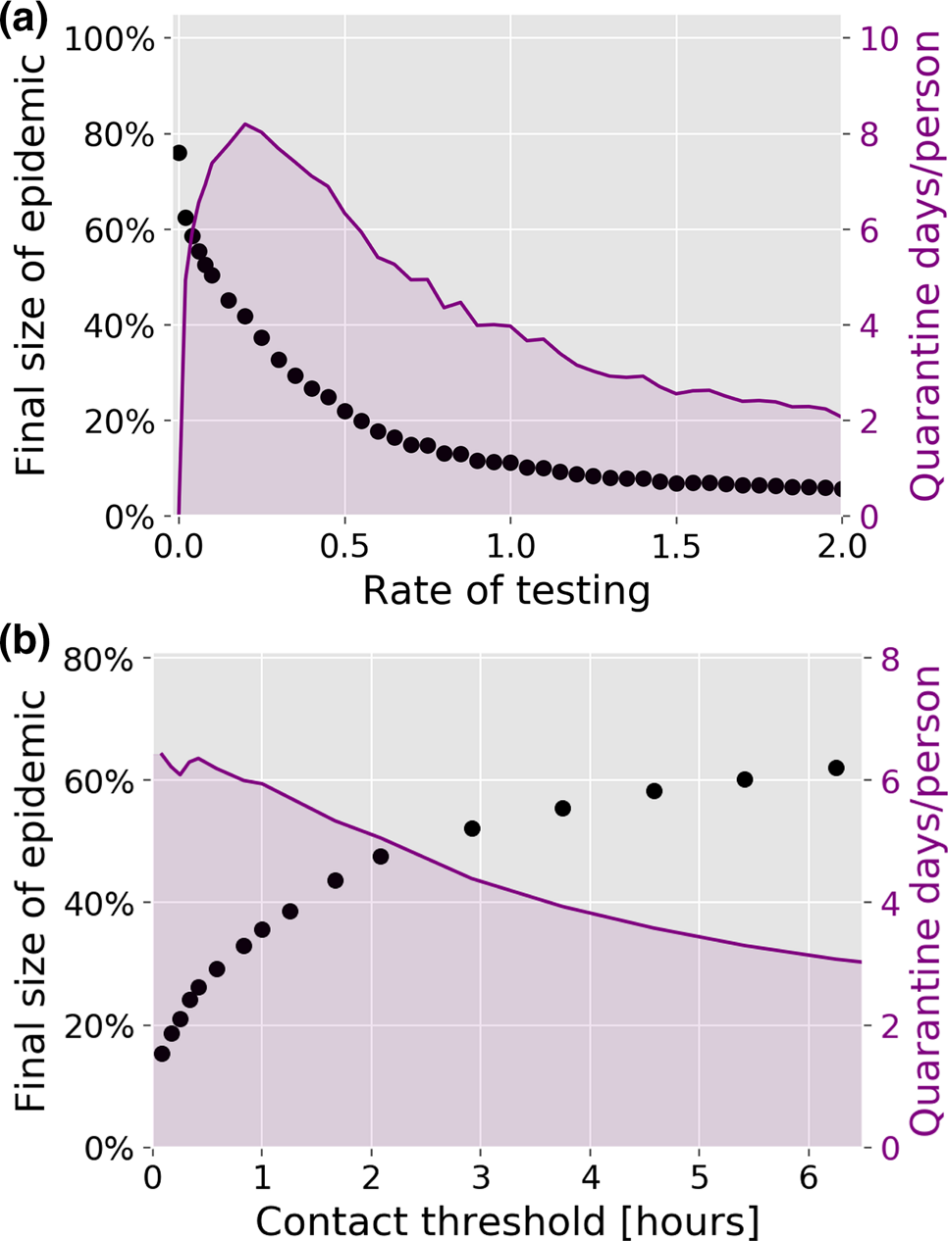
Contact tracing effectiveness. Disease parameters are identical to those of Fig. 3. **a** Rate of testing vs final size of epidemic and average number of days spent in quarantine per person. The contact threshold is set at 15 min. The rate of testing is measured in units of the rate for leaving the infected state, meaning that a rate of testing of 1 corresponds to a 50% chance of being tested during the infectious period. **b** Contact threshold vs final size of epidemic and average number of days spent in quarantine per person. The rate of testing is set at 0.5 times the rate for leaving the symptomatic infectious stage, giving a 25% probability of being tested while infected. For each value of the parameter, 50 simulations were run

For contact tracing to be effective at higher contact thresholds, a substantial degree of temporal correlation in contact dynamics is necessary. Both edge swapping and randomization reduce the temporal correlation. To quantify this, we find that the median fraction of long contacts (of at least 60 min cumulative duration) which are repeated from 1 week to the next is 30% in the real network, while edge swapping and randomizing reduces this number to zero. Evidently, repeated contacts are necessary for tracing to be effective at higher thresholds.

The edge swapping procedure reduces temporal correlation in two ways, which may be studied separately. Firstly, it destroys correlations in social structure (“who meets who”), i.e. the identities of contact partners are randomized, reducing the occurrence of repeated contacts as described above. Secondly, the procedure destroys the duration distribution of contact *durations*, breaking e.g. a 10 min contact into two uncorrelated 5 min contacts. In the Supplemental Material, we describe an alternative edge swapping algorithm which preserves the durations of contacts, while still swapping the individuals. This allows us to study the importance of the contact duration distribution and the existence of repeated contacts separately. At high contact tracing thresholds (125 min) We find that even duration-preserving edge swapping reduces the mitigative effect of contact tracing relative to the true network. The reduction is not as strong as with the simple edge swapping algorithm, leading us to conclude that there are two effects at play: destroying the duration distribution leads to poorer performance, but simply randomizing the identities of contacts while preserving degree and duration distributions has a detrimental effect in and of itself. Conversely, one may conclude that repeated contacts (temporal correlations in the identities of contact partners) as well as an inhomogeneous contact duration distribution are important features which improve the effectiveness of contact tracing. Our finding that contact tracing on the randomized network performs poorer than the edge swapped version is in good agreement with the findings of [51], which shows theoretically that the presence of highly connected *hubs* in a social network improves contact tracing.

Due to the limited temporal resolution of the Bluetooth proximity data, obtained only at 5 min intervals, the fidelity at very short contact tracing thresholds of e.g. 5 min will be lower. In the Supplemental Material we explore the results obtainable at these lower contact thresholds, and present a theoretical argument for the observed patterns. We find that mitigation by test-trace-isolate (TTI) is always more *efficient* on the true network, in the sense of preventing more cases per day of quarantine, but that the randomized network may in fact lead to a *lower* final attack rate when contact thresholds tend to zero. The effect is due to the number of quarantines triggered in the random network diverging in this limit.

*The rate of testing and contact threshold* The regular testing considered in our contact tracing algorithm is determined by a rate of testing which reflects several real-world factors not individually modeled here, such as general test capacity, symptom development and willingness to participate in testing. In Fig. 5a, we explore the influence of the rate of testing on the final size of the epidemic and the average time spent in quarantine. As one would expect, the quarantine time vanishes at very low rates of testing, where the epidemic size is maximal. Whereas the epidemic size is a decreasing function of testing, the quarantine time does not display a simple monotonic response to an increase in testing. Rather, it attains a maximum at 10% probability of being tested, followed by a gradual decline. This highlights the importance that changes in the testing strategy should go hand-in-hand with considerations of the nontrivial influence on the quarantine time. As such, it is possible to achieve a lower total quarantine time by increasing testing levels, simply due to the improved epidemic control.

If the aim is to keep the final size of the epidemic below for example 25%, our results show that a contact threshold of less than 30min is necessary (Fig. 5b). Note that the concurrent implementation of other mitigation strategies such as social distancing or limits on gathering will increase this critical threshold.

## Discussion

In order to assess and credibly model the effectiveness of mitigation strategies, it is necessary to know which idealizations can be safely made, and which complexities must be retained in models. The present work shows that realistic social structure is an indispensable complexity when attempting to model contact tracing strategies and predict their effectiveness.

Although the social proximity data used in this study do not represent the social activity in a complex society, it exhibits a relevant level of social heterogeneity, which is stable over timescales long enough that it can influence epidemic dynamics. In this sense, the data can serve as a valuable model system in which to evaluate the impact of heterogeneity on disease propagation and mitigation of epidemics. We have found that social activity levels are exponentially distributed in this cohort, something which is consistent with observations of [7], who find a coefficient of variation of 0.8 for social contacts, for persons aged 20–30. The person-specific social activity exhibited in our data remains consistent over time, with both the 1-day and the 7-week activity patterns having coefficients of variations close to 1, representing a quenched disorder on the relevant timescale.

Even in the absence of mitigation, the social heterogeneity exhibited by our cohort significantly affects the epidemic trajectory. However, not all outcomes are affected similarly. The epidemic peak height is found to be sensitive to the social structure, while the final size of the epidemic is primarily affected by heterogeneity in social *time*. The isolated sensitivity of attack rates to heterogeneous social activity, i.e. differences in contact *time*, can be studied in a well-mixed compartmental model, as was recently done [52], underscoring that the effect is not only present in structured networks. The influence of social structure, however, is a more complex phenomenon and requires network models, either synthetic or using observational social network data [1]. The effects of network structure in epidemic spreading were previously studied by Barthélemy et al. using synthetic social networks, which were however assumed static [50]. They found the mechanism to be a hierarchical progression, with more well-connected individuals being infected early on, and more sparsely connected nodes being affected later in the epidemic, if at all. However, this mechanism depends on connectivity being a *quenched* variable, i.e. one that sticks to each individual over time. We find that this condition is satisfied, at least on a timescale of a few months.

The sizable effects of social structure and heterogeneous activity seen in this study has implications for epidemiological modelling in general. Due to their lack of social structure, traditional well-mixed S(E)IR models would overestimate the severity of the epidemic, or, conversely, lead to an underestimation of transmission risk when fitted to an observed epidemic trajectory. In a previous modelling study [53], it was shown that heterogeneity in the *susceptibility* of individuals likewise reduces the overall severity.

Once contact tracing is implemented, the effect of social heterogeneity becomes more complex. We found that social structure and heterogeneous activity levels substantially increase the efficiency of contact tracing. However, when the contact tracing threshold is low, heterogeneity in activity levels alone improves effectiveness substantially, and network structure alone has less of an effect. When the contact tracing threshold is high, both social network structure and heterogeneous activity levels are necessary for efficient tracing. Furthermore, we found that the presence of heterogeneity in contact duration improves the efficiency of contact tracing in itself. Our findings also highlight that neither a quenched nor an annealed view of contact networks are sufficient for modelling the spread of a disease such as COVID-19, since no clear separation of scales is present. Important network dynamics takes place on time-scales shorter than an infectious period, while some aspects of network structure and social activity are stable on timescales corresponding to several generations of the disease. While many previous approaches have relied on such a separation of time scales, sophisticated analytic frameworks for epidemic spreading on time-varying networks have been proposed in recent years, allowing for e.g. continuously varying networks [54, 55].

The two central parameters of our contact tracing algorithm, the *rate of testing* and the *contact threshold*, are not on equal footing. The rate of testing is influenced both by factors which are within our control, such as the overall availability of testing, and by factors which are essentially intrinsic to SARS-CoV-2, such as the rate at which symptoms develop. The contact threshold, on the other hand, is a fully controllable parameter and essentially constitutes a design decision when e.g. developing contact tracing applications [35]. Our results indicate that the contact threshold must be kept quite low ($<30$ min) if relatively efficient control (reducing epidemic final size by about two thirds) is to be attained in an otherwise unmitigated epidemic. We find that the strength of mitigation depends strongly on the rate of testing. This is expected since the ability to trace contacts depends on the chance of identifying at least one case in the infection chain by regular testing. What is perhaps less obvious is that the total quarantine time has a nontrivial (inverted U-shaped) dependence on the rate of testing. As the rate is increased from 0, the quarantine time increases. However, once an appreciable level of epidemic control has been achieved through contact tracing, it begins to decline, with the peak value being attained at a rate corresponding to a 10% probability of being tested while infected.

In this study, we have only considered forward contact tracing, where the primary objective is to track down individuals who might have been infected by the index case. However, other schemes exist, and two recent papers which came out after the initial publication of this manuscript have shown that backwards contact tracing has an advantage in scenarios with highly clustered cases [56, 57], i.e. where the transmission dynamics is overdispersed such that a few individuals cause a high number of secondary infections while the majority cause few. Such clustering may arise by several mechanisms of biological as well as social origin [58]. Recently, several studies have found that COVID-19 transmission is in fact highly heterogeneous [58–64]. While we have focused on the impact of social heterogeneity on mitigation by contact tracing, a recent study showed that heterogeneity in biological infectiousness has a considerable impact on the feasibility of COVID-19 mitigation strategies which rely on contact network reduction [65], such as lockdowns.

While our study has highlighted the importance of network and activity heterogeneity for the efficiency of contact tracing, some previous studies have highlighted other network measures, such as *degree*, *betweenness* and *reach* as useful in further targeting contact tracing [30, 66]. It is our opinion that there is still much to be learned about the usage of network data for the improvement of contact tracing – and in order to identify the relevant mechanisms, modeling studies are indispensable.

In conclusion, heterogeneity in social activity makes mitigation by contact tracing much more effective. If only more frequent contacts can be traced, social network structure becomes important as well. It is thus important that realistic social heterogeneity and structure be taken into account when modeling contact tracing, as failure to do so may lead to underestimation of its effectiveness.

## Supplementary Information

Below is the link to the electronic supplementary material.

Supplementary material 1 (pdf 2475 KB)

Supplementary material 2 (pdf 49 KB)

Supplementary material 3 (csv 78648 KB)

## Data Availability

All data analysed during this study are included in the supplementary information file “data.csv”. The simulation code developed during the study is available in the *EmpiricalNetworksCT* GitHub repository, https://github.com/NBIBioComplexity/EmpiricalNetworksCT/.
