# Supplementary material for: Differences in social activity increase efficiency of contact tracing
Source: Eur Phys J B. 2021 Oct 19;94(10):209. doi: 10.1140/epjb/s10051-021-00222-8 (PMC8523203; doi:10.1140/epjb/s10051-021-00222-8)
Supplement: Supplementary file 1 — Supplementary material 1 (pdf 2475 KB) [file 10051_2021_222_MOESM1_ESM.pdf]

Supplemental Material: Differences in social activity increase efficiency of contact tracing

Bjarke Frost Nielsen,[1] Kim Sneppen,[1] Lone Simonsen,[2] and Joachim Mathiesen[1]

[1]*Niels Bohr Institute, University of Copenhagen, 2100 Copenhagen, Denmark*

[2]*Department of Science and Environment, Roskilde University, 4000 Roskilde, Denmark*

(Dated: August 4, 2021)

I. THE DURATION-PRESERVING EDGE SWAPPING ALGORITHM

In this section we explore the results of the duration-preserving edge swapping algorithm mentioned in the main text, as well as the algorithm itself.

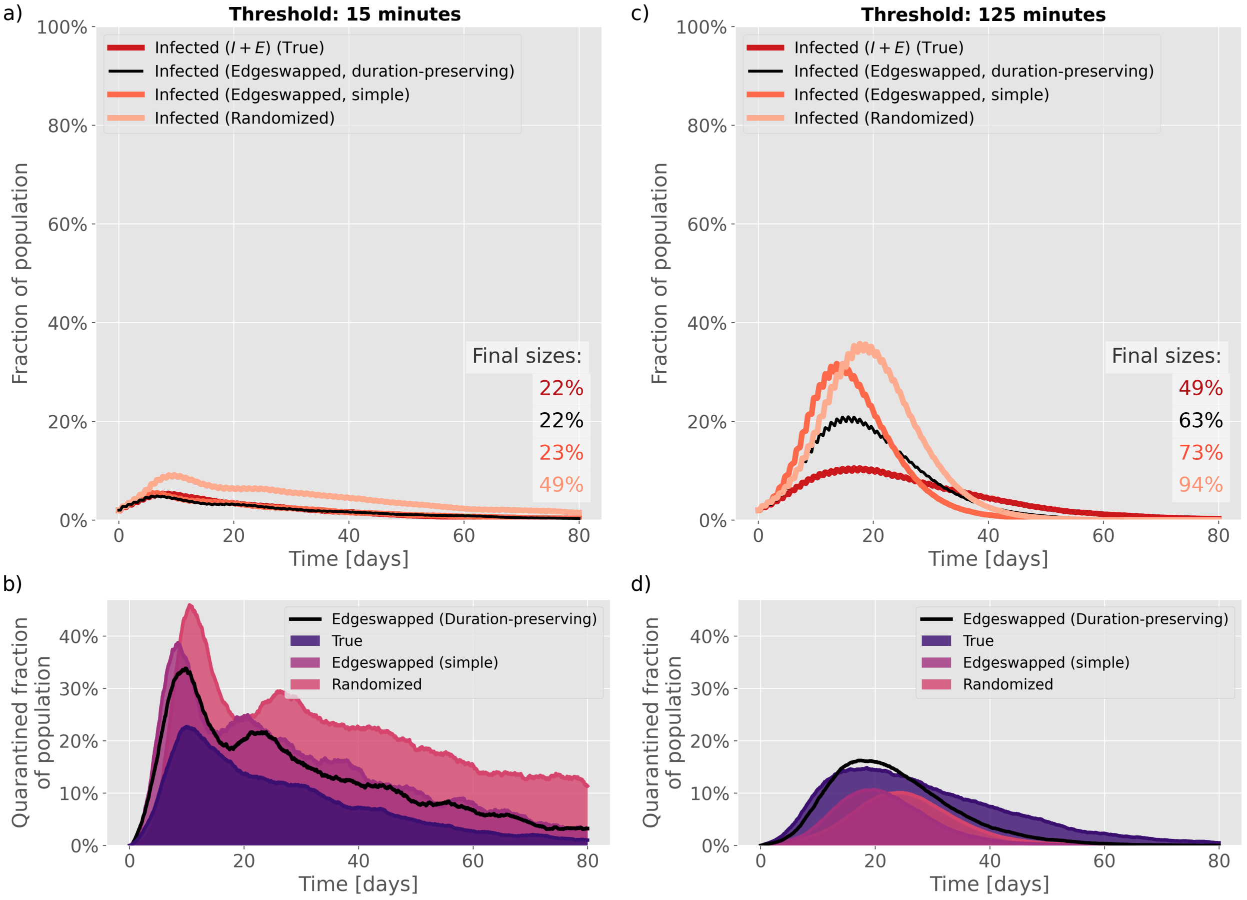

FIG. S1. **Duration-preserving edge swapping: effects on contact tracing at different thresholds.** Companion figure to Fig. 4 of the main text. The red curves show the trajectories of the outbreaks in the three networks types described in the main text (true, edge swapped and randomized) while the black curves show the corresponding results for the duration-preserving edge swapping algorithm. The testing rate is set at 0.5 times the rate for leaving the symptomatic infectious stage, giving a 25% probability of being tested while infected. **a)** Number of infected ($I + E$ states, including presymptomatic) with a contact tracing threshold of 15 minutes. **b)** The fraction of the population under quarantine as a function of time, with a contact tracing threshold of 15 minutes. **c)** Same as panel a, but with a contact tracing threshold of 125 minutes. **d)** Same as panel b, but with a contact tracing threshold of 125 minutes.

We begin by presenting the results of this version of edge swapping. The duration-preserving edge swapping algorithm proceeds as follows:
We refer to the collection of fixed-time social networks at time steps $t = 1, 2, \ldots, T$ as $G = \{G_t\}$ where G_t is the network of (Bluetooth) contacts at time step t, representing a 5 minute window determined by the temporal resolution of the experimental data.

To prepare for edge swapping, we first run through the graph collection G, recording at which time step each contact begins, its duration and a persistent contact ID, allowing recognition of equivalent edges across several time steps. The edge swapping itself then proceeds similarly to the simple (duration non-preserving) version, with a few modifications:

- For $t \in \{1, \ldots, T\}$:
  - For each edge in G_t, check whether it corresponds to a contact which began at time t (i.e. the contact has just been initiated). If this is the case, add the ID of this contact to a list $L = \{L_i\}$.
  - Once the list L of newly-initiated contacts has been compiled, perform a random pairing of IDs, stratified by duration, such that only contacts of equal duration are paired.
  - Perform the simple edge swapping algorithm described in the main text, but only within each group of equal-duration contacts.
  - Record all swaps performed within this time step, such that they can be consistently applied in the succeeding time steps (for as long as each swapped contact lasts)
  - Perform swaps which were recorded in preceding time steps, unless the duration of the contacts has elapsed (in which case: discard the swap).

It is also possible to devise edge swapping algorithms in which durations are *binned*, such that contacts of *approximately* equal duration are swapped. However, it is not possible to do this without either altering the time-development of overall social activity (number of edges active in a certain time step) or the contact duration distribution for each individual.

In Fig. S1, the epidemic trajectories of an outbreak with contact tracing is shown for a social network which has undergone this duration-preserving edge swapping procedure. The results are compared to those of the "usual" true, edge swapped and randomized networks

of the main text. At a (high) threshold of 125 minutes, the duration-preserving network performs intermediately, with the final size of the epidemic being between those of the true and edge swapped networks. In the case of a (short) contact threshold of 15 minutes, the difference between true, edge swapped and duration-preserving edge swapped is negligible in terms of final size, but the number of quarantines issued differs substantially. For comparison, the final epidemic size in the duration-preserving edge swapped network was found to be 79% in the completely unmitigated scenario. This figure is quite similar to the 76% and 80% measured in the true and edge swapped networks, while being much lower than the 98% obtained for the randomized network, reflecting the finding of the main text that the unmitigated final size depends strongly on a heterogeneous degree distribution.
In the next section, we quantify the reduction in final size *per* quarantine in each of the scenarios as a measure of the *efficiency* of the combined mitigation strategy, consisting of regular testing and contact tracing.

II. PREVENTED CASES PER QUARANTINE

The TTI (test-trace-isolate) mitigation strategy employed in this paper relies on quarantine and has the time spent in quarantine as a major societal cost. In this section, we attempt to quantify the efficiency of such a strategy by computing the number of cases *prevented* by each week of quarantine, for each of the four network types - True, Edge swapped, Randomized and the Duration-preserving edge swapped version introduced in the previous section.
The results are summarized in Table I, which shows that each quarantine issued in the True network prevents more transmission than a quarantine issued in the other network types. Measured in this way, the mitigation strategy is successively less efficient in the Duration-preserving, Edge swapped and Randomized networks, showcasing how each additional layer of heterogeneity improves the efficiency of the mitigation strategy.

III. VERY SHORT CONTACT THRESHOLDS

In the main text, we probe only contact thresholds ≥ 15 minutes. This choice was made for two reasons: real-world practicality and the limited temporal resolution of the

Network	Attack Rate (AR) (15 min.)	$\Delta(AR)/Q$ (15 min.)	AR (125 min.)	$\Delta(AR)/Q$ (125 min.)
True	22%	0.60	49%	0.37
Duration-preserving e.s.	22%	0.39	63%	0.27
Edge swapped	23%	0.35	73%	0.22
Randomized	49%	0.21	94%	0.14

TABLE I. Mitigation efficiency measured by final attack rate (abbreviated as AR) and cases prevented per week of quarantine per person quarantined, abbreviated as $\Delta(AR)/Q$. The first two data columns refer to simulations with a contact threshold of 15 minutes, while the last two data columns refer to simulations with a contact threshold of 125 minutes.

empirical data used in this study. When contact thresholds are very short, the number of 'problematic contacts' generated by each new infection diverges, leading to issues of practicality. Furthermore, any 5 minute (and, to a lesser extent, the 10 minute) simulations will have some inherent limitations due to the way the Bluetooth proximity data in this study was obtained. Since the Bluetooth ports of all the participating devices were open for scanning and discoverability once every 5 minutes, this is the shortest duration that we can resolve. This also means that even two persons passing each other just as the Bluetooth ports are active will show up as a 5 minute contact. Nonetheless, we here include some results of 5 and 10-minute simulations here to showcase some tendencies which become clear at very low contact thresholds.

With the help of a thought experiment, one may convince oneself that in the limit of infinitely short contact threshold, the edge swapped and randomized algorithms should lead to more cases prevented by contact tracing, by virtue of each person coming into contact with a higher number of different individuals. However, this increased mitigation effect occurs at the cost of diverging quarantine numbers.

In an idealized situation with an arbitrarily fine-grained temporal resolution ($\Delta t \rightarrow 0$), randomization as well as edge swapping will lead to an "everyone meets everyone" situation (even if some individuals have more contact time than others in the edge swapped scenario). If the contact threshold then also tends to zero, any infected individual who is discovered by regular testing will trigger a quarantine for everyone. For the duration-preserving edge

Network	Attack Rate (AR) (5 min.)	$\Delta(AR)/Q$ (5 min.)	AR (10 min.)	$\Delta(AR)/Q$ (10 min.)
True	16%	0.64	19%	0.62
Duration-preserving e.s.	13%	0.49	17%	0.45
Edge swapped	11%	0.49	16%	0.43
Randomized	8%	0.57	22%	0.34

TABLE II. Mitigation efficiency measured by final attack rate (abbreviated as AR) and cases prevented per week of quarantine per person quarantined, abbreviated as $\Delta(AR)/Q$. The first two data columns refer to simulations with a contact threshold of 15 minutes, while the last two data columns refer to simulations with a contact threshold of 125 minutes.

swapping algorithm, this effect is not quite as strong, since longer contacts are not 'broken down' into several shorter ones by the swapping algorithm (but if several longer contacts between the same two persons exist, these will still be 'swapped' with multiple different persons).

In Table II we present results on the efficiency of mitigation in each of the four network types, analogous to those presented in Table I, but for the shortest possible contact thresholds of 5 and 10 minutes. Measured in this way, it is clear that the true network always leads to the highest efficiency - even if it does not lead to the largest reduction in the 5 minute case, it obtains the reduction with by far the lowest number of quarantine days. In Fig. S2 we show the epidemic trajectories and quarantine curves obtained for the 5 and 10 minute contact threshold simulations. The results confirm the intuition from the thought experiment, that at sufficiently low contact threshold the number of persons placed in quarantine in the edge swapped simulations becomes so large that the total epidemic size actually becomes smaller than for the true network.

IV. THE CONTACT RETENTION TIME

In our contact tracing scheme, information about encounters are only kept for a certain time. When a person is tested positive, all recorded contacts older than this *retention time* are deleted, so as to not cause an inordinate amount of quarantines of individuals who are

at very low probability of having been infected. We here present a sensitivity analysis of our results with respect to this parameter. In the main text, a retention time of 5 days is used. This parameter always represents a trade-off, with high values giving a high probability of finding infected contacts, but also causing a large number of unnecessary quarantines. Low

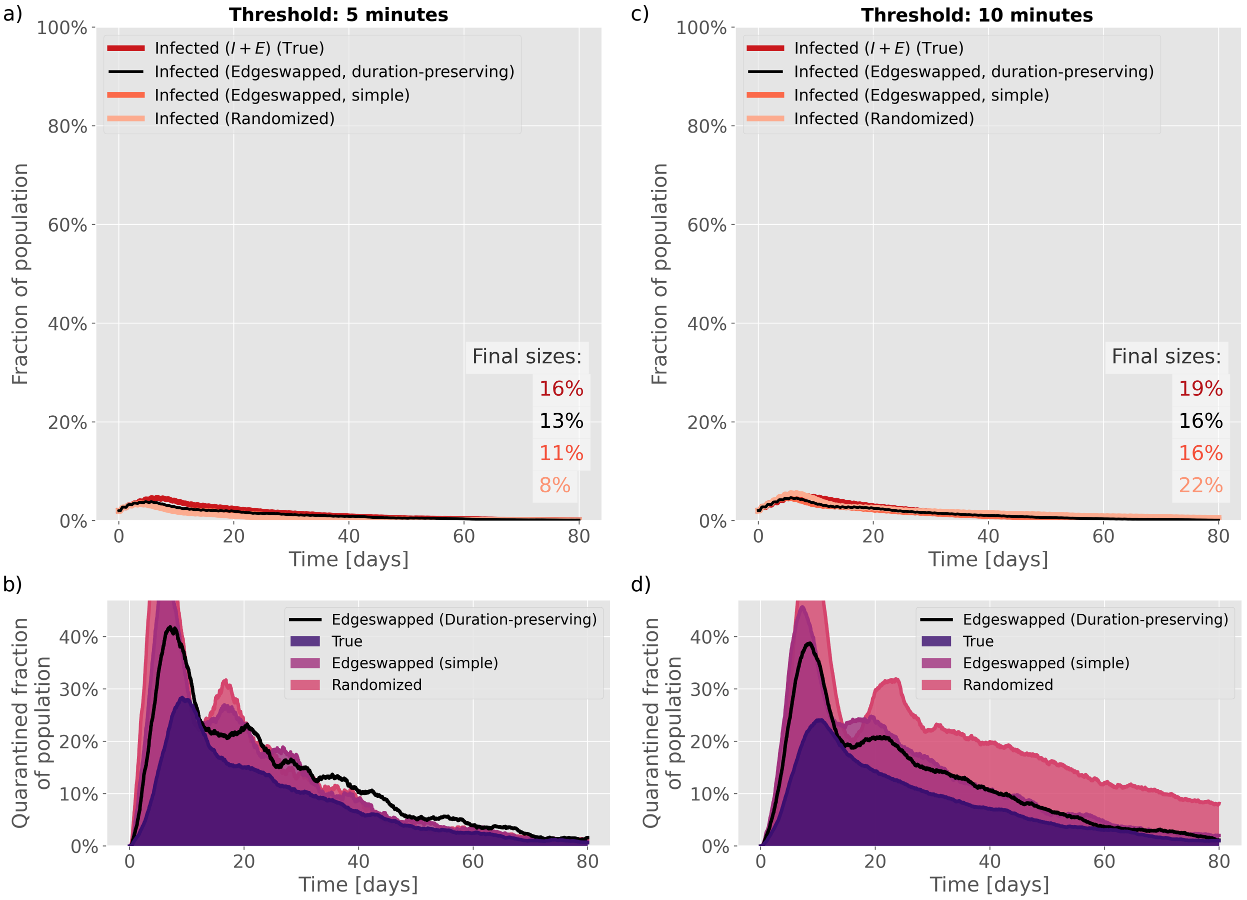

FIG. S2. **Contact tracing at very low thresholds** Companion figure to Fig. 4 of the main text. The red curves show time development of the outbreak in the three networks types described in the main text (true, edge swapped and randomized) while the black curves show the corresponding results for the duration-preserving edge swapping algorithm. The testing rate is set at 0.5 times the rate for leaving the symptomatic infectious stage, giving a 25% probability of being tested while infected. **a)** Number of infected ($I + E$ states, including presymptomatic) with a contact tracing threshold of 5 minutes. **b)** The fraction of the population under quarantine as a function of time, with a contact tracing threshold of 5 minutes. **c)** Same as panel a, but with a contact tracing threshold of 10 minutes. **d)** Same as panel b, but with a contact tracing threshold of 10 minutes.

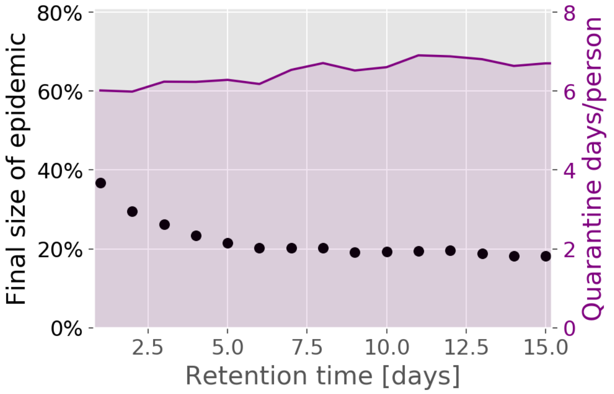

FIG. S3. **The effect of the retention time on contact tracing-based mitigation.** The purple curve shows the average number of days that a person spends under quarantine during the entire epidemic. The black makers show the final size of the epidemic, defined as the fraction of the population which is exposed to the disease at any point during the epidemic. This parameter sensitivity analysis was run with a minimum quarantine duration of 5 days, a contact tracing threshold of 15 minutes and a testing probability of 25%.

values decrease epidemic control while also reducing the quarantine overhead. The precise relationship is shown in Fig. S3. We find that a retention time of 5 days is reasonable, since the attack rate decreases only very slowly after that point. Overall, the total time spent in quarantine increases with increasing retention time, as expected, but the dependence is only moderate.

V. THE (MINIMUM) QUARANTINE DURATION

Once a contact of an infected person is identified via contact tracing, they are placed into quarantine. This quarantine has a minimum duration, to prevent release of exposed individuals who have not yet converted. As shown in Fig. S4, a very long minimum quarantine duration leads to a high degree of epidemic control while also causing the average person to spend a more days in quarantine. When quarantine duration is set at less than 5 days, the marginal decrease in attack rate upon increasing the duration is sizable. The curves flattens somewhat after this point, and a minimum quarantine duration of more than 9 days confers little benefit.

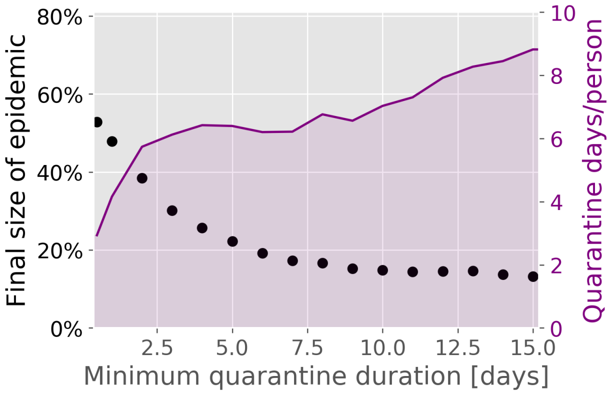

FIG. S4. **The effect of the minimum quarantine duration on contact tracing-based mitigation.** The purple curve shows the average number of days that a person spends under quarantine during the entire epidemic. The black makers show the final size of the epidemic, defined as the fraction of the population which is exposed to the disease at any point during the epidemic. This parameter sensitivity analysis was run with a retention time of 5 days, a contact tracing threshold of 15 minutes and a testing probability of 25%.
