# Supplementary material for: Differences in social activity increase efficiency of contact tracing
Source: Eur Phys J B. 2021 Oct 19;94(10):209. doi: 10.1140/epjb/s10051-021-00222-8 (PMC8523203; doi:10.1140/epjb/s10051-021-00222-8)
Supplement: Supplementary file 2 — Supplementary material 2 (pdf 49 KB) [file 10051_2021_222_MOESM2_ESM.pdf]

The supplementary data file, data.csv, is a comma separated file containing the data used in the manuscript. We cannot release the full data set due to privacy concerns. The data set however contains all the relevant information (approximately 4 million events) to reproduce the main findings of the manuscript. The file consists of 5 columns, where the first is a timestamp in seconds since the beginning of a week. The second is the id of the user. The third column contains the id of other users in the proximity, where proximity is measured by rssi value in the fourth column. Finally, the 5th column is an identifier of a week. Note that weeks are not consecutive but chosen randomly from the full data set.
